# Analysis of the *Agger nasi cell* and frontal sinus ostium sizes using computed tomography of the paranasal sinuses

**DOI:** 10.5935/1808-8694.20130052

**Published:** 2015-10-04

**Authors:** Fernando Veiga Angélico Junior, Priscila Bogar Rapoport

**Affiliations:** a MSc in Health Sciences; Assistant Professor and Coordinator of the Medical Residency Program in Otorhinolaryngology of the Medical School of the ABC.; b PhD in Otorhinolaryngology; Full Professor of Otorhinolaryngology - Medical School of the ABC. Medical School of the ABC (FMABC).

**Keywords:** anatomy, ethmoid sinus, frontal sinus, measures, tomography

## Abstract

The *Agger nasi cell* (ANC) and the frontal sinus ostium (FO) are important structures that can influence the anatomy and physiology of the frontal recess. The aim of this study was to evaluate the presence and size of ANC and the FO and correlate them according to gender, race and among themselves.

**Method:**

A prospective study with 40 patients who underwent CT of the paranasal sinuses with sagittal reconstruction. Measurements: ANC (APAN) anteroposterior diameter, ANC (CCAN) craniocaudal diameter, ANC (LLAN) side-to-side diameter, anteroposterior diameter of the FO (APFO) and side-to-side diameter of the FO (LLFO).

**Results:**

Twenty-two patients were male and 18 females, mean age 33.7 years. Most patients were white (45%), followed by browns (32.5%), blacks (20%) and asians (2.5%). The ANC was present in 98.7% of patients. There was statistical difference for APAN on females and LLAN on females and on the total sample. There were no differences for all measurements regarding gender, as well as the race. ANC and FO measurements showed positive correlation, but poor or very poor.

**Conclusion:**

The prevalence of ANC in our sample was high and did not show a statistically significant difference for most measurements. The correlation between measurements of ANC and the FO was poor or very poor.

## INTRODUCTION

The frontonasal region anatomy acquired much importance, mainly after the development of techniques for approaching this region through nasal endoscopy.

In recent years, endoscopic endonasal surgery (EES) has been widely used for the treatment of the most varied sinonasal disorders, especially of the frontal sinus (FS)^1^, ^2^. However, the frontal endoscopic sinusotomy remains a challenge for most otorhinolaryngologists, due to the complexity and anatomical variability of the three-dimensional spaces called ethmoidal infundibulum and frontal recess (FR)^3^, ^4^, ^5^.

The FR is the anterosuperior border of this complex, being the embryological origin of the FS. Its medial border is the lateral surface of the anterior portion of the middle concha, all the way to its insertion in the skull base. Should the unciform process curve medially and inserts in the middle concha, it will also be part of the FR medial border. The lamina papyracea is the lateral recess border, and the unciform process may be part of such border when it is inserted superiorly or laterally to it. The posterior boundary is created from the anterior surface of the ethmoid bulla, which is typically inserted at the skull base, which may be an incomplete insertion. The anterior border includes the *Agger nasi* (AN), which can be pneumatized and of varied size. When the ANC is pneumatized, we have the *Agger nasi cell* (ANC) formation^6^, ^7^, ^8^.

In the sagittal plane, the frontonasal communication takes the form of an hourglass, which more closely corresponds to the frontal sinus ostium (FO)^9^, ^10^. Below the FO the FR dimensions are determined by several structures which also contribute to its physiological functioning.

This anatomical pattern may be influenced by anterior ethmoidal cells that develop embryologically around the FR, which could have profound anatomical and functional repercussions on the frontonasal communication^11^.

Therefore, any malformation or existing anatomical variation in this area can affect FS drainage and ventilation, causing difficult-to-treat rhinosinusitis. Several factors can narrow the FR, especially the ANC, the ethmoidal bulla, the unciform process and even the middle concha head^9^, ^12^, ^13^. Thus, surgery aiming at eliminating a FR disorder or to correct an anatomical alteration or malformation will lead to restoration of normal FS function, due to improved ventilation and drainage^11^.

With the development of computed tomography (CT) and fiber optic systems, more data can be obtained in the preoperative period, aiming at a better treatment choice. The critical analysis of these data, associated with intraoperative findings are important in the decision to do a more extensive approach on the ethmoidal infundibulum and FR, sometimes leading to a resection of the thick bone adjacent to the FO.

The paranasal sinuses computed tomography scan (PSCT) is the method of choice for image evaluation of nasal and sinonasal diseases and to study the ostiomeatal complex^14^, ^15^.

Usually, there are two views: coronal and axial. These views may bring detailed information about FS size, ANC size and ethmoidal infundibulum structures that can compromise and alter the FR.

The sagittal reconstruction, with fine slices used in recent years, allows for a better analysis of nasal structures and brings a fresh push to understanding the complex anatomy, allowing us to measure the sizes of structures that make up or contribute to the FR formation^9^, ^16^, ^17^. It also allows the identification of the anteroposterior relationship between the FR, the unciform process, ethmoidal bulla, basal lamella of the middle concha and the upper concha, which can be difficult to assess only under nasal endoscopy or conventional CT scan.

Thus, the study of the FR region is of fundamental importance in the management of frontal rhinosinusitis.

Thus, the objectives of this study are: to evaluate the prevalence of the *Agger nasi cell* (ANC), measure the dimensions of the ANC and the frontal sinus ostium (FO), compare the dimensions of the ANC and FO in both genders and between the races, and check the relationship of the ANC and FO dimensions.

## METHOD

We consecutively included volunteer adults, of both genders, coming from the general outpatient clinic of a tertiary hospital, who required PNSCT to investigate frontal headache with or without other nasal symptoms.

The study protocol was reviewed and approved by the Ethics in Research Committee of the institution under number 102/2003, and all patients signed an informed consent form.

All patients were evaluated, obtaining data from clinical history, general physical examination and ENT physical exam. They were also submitted to nasofibroscopy with a 4 mm 0 degree hard endoscope (Fiegert Endotech brand).

The TCSP was performed in the same hospital and all the images were acquired using a GE scanner CT/and helical (GE Medical Systems, Milwaukee, Wisconsin, USA) in the axial plane, with the patient in the supine position with the head set at a neutral position. We used the helical volumetric acquisition technique without angle, with contiguous 1 mm thick slices at 1 mm interval; parameters: 120 kV, 60 mA, 1s/rotation) in bone window (HU-4000 level - Hounsfield Units), stretching from the nasal process of the maxilla to the apex of the frontal sinus, parallel to the hard palate. From the acquired images, we made a reconstruction of the coronal and sagittal planes on the workstation installed on the scanner and the recording was made in discs for later analysis.

The exclusion criteria were: patients under the age of 18 years; absence of frontal sinus; presence of nasal-related headache, chronic rhinosinusitis, sinonasal polyposis, bone erosions, sinonasal tumors, anatomical malformations of the paranasal sinuses or any other condition that prevents the visualization of the bony structures of the region studied; presence of ethmoidofrontal cells, frontal intersinusal cells, supraorbital ethmoidal cells, supra bullae cells, fontal bulla or any other anatomical variation close to or obstructing the FR or FO, altering its dimensions; any CT alteration which prevents measurements of the ANC and/or the FO, such as, for example, artifacts caused by dental restorations; and those who refused to sign the Informed Consent Form.

We analyzed 80 nasal cavities, corresponding to 40 patients, evaluating the axial, coronal and sagittal vies.

For FO identification we initially used the sagittal views, where it is easier to pinpoint the internal nasal spine, the FR and the FO. On the CT scanner workstation we could see, at the same time, the axial, coronal and sagittal views in one same screen and, moving the marking cursor in order to measure the structures on the image in one determined type of view, the cursor moves automatically in the others two views, simultaneously. So, we were able to identify and more accurately measure the dimensions of the structures under consideration. Similarly, we pinpointed the ANC and measured it.

**Figure 1:**
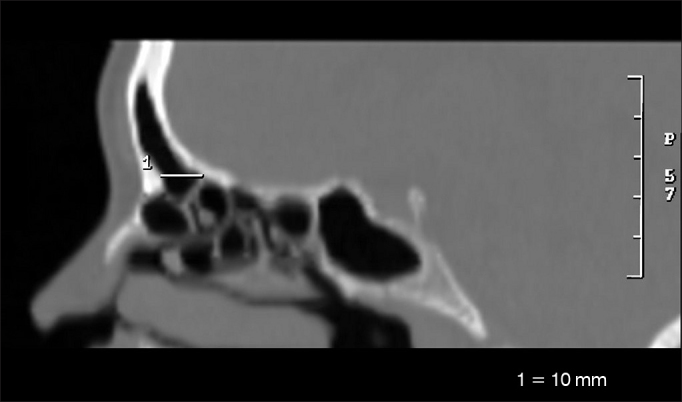
Anteroposterior measure of the frontal sinus ostium (OFAP).
• Side-to-side diameter of the frontal sinus ostium (OFLL) (Figure 2)

The following measures were taken:

• Larger anteroposterior diameter of the frontal sinus ostium (OFAP) (Figure 1)

**Figure 2:**
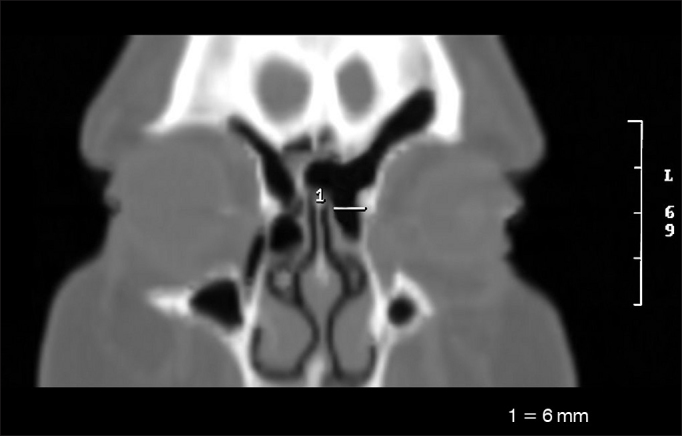
Side-to-side measure of the frontal sinus ostium (OFLL).
• Larger anteroposterior diameter of the *Agger nasi cell* (AGAP) (Figure 3)

**Figure 3:**
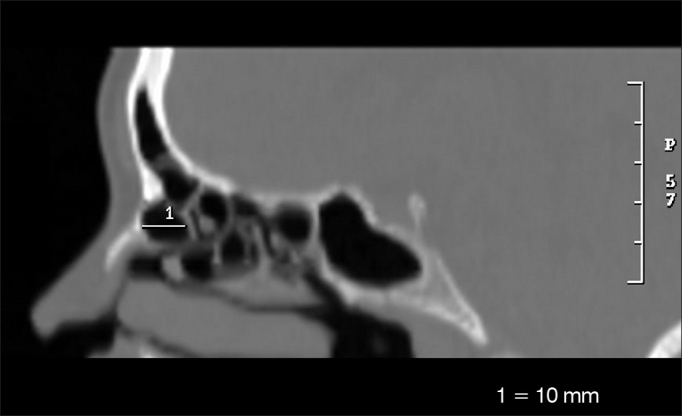
Anteroposterior measure of the *Agger nasi cell* (AGAP).
• Larger side-to-side diameter of the *Agger nasi cell* (AGLL) (Figure 4)

**Figure 4:**
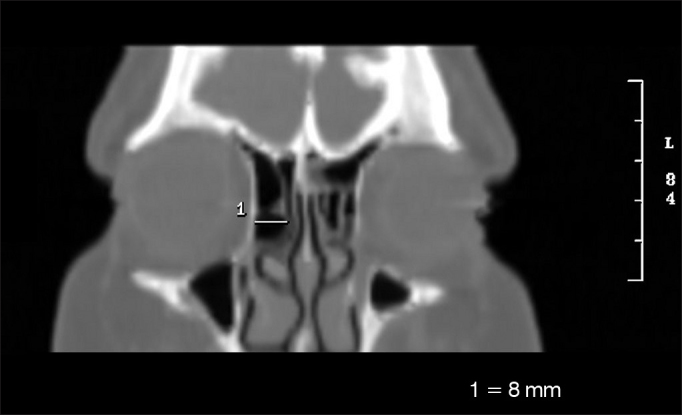
Side-to-side measure of the *Agger nasi cell* (AGLL).
• Larger craniocaudal diameter of the *Agger nasi cell* (AGCC) (Figure 5).

**Figure 5:**
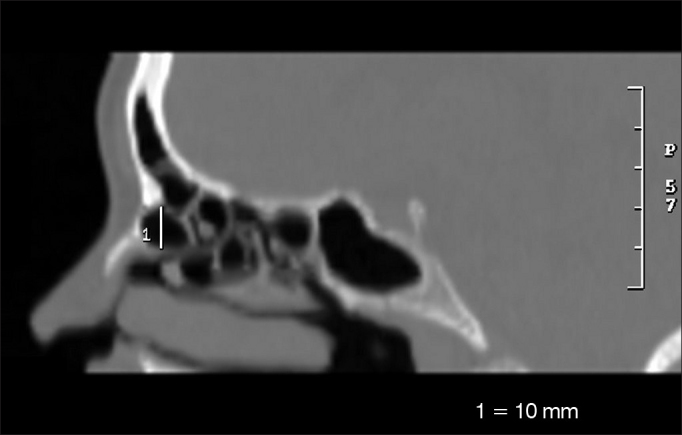
Craniocaudal measure of the *Agger nasi* cell (AGCC).

For the statistical analysis, we used the following programs: SPSS V16 (Statistical Package for Social Sciences - version 16.0) 15, Minitab and Excel Office™ to obtain the results. We used the following tests: ANOVA (Variance Analysis), *Student's* paired *t*-test and the test for equality of two ratios to assess FO and ANC measures and the Pearson's correlation coefficient in order to establish possible relationships between the variables above.

For all analyses, we considered the 95% confidence interval and 5% significance interval (*p* < 0.05).

## RESULTS

Our series comprises 40 patients, 18 (45%) females and 22 (55%) males, with no statistical difference between them (*p* = 0.371).

The patient's age range varied between 19 and 54 years, averaging 33.7 years and with a standard deviation of 9.7 years. There was no statistically significant difference between the genders vis-à-vis age (*p* = 0.922).

Most patients were caucasian (45%), followed by browns (32.5%) and blacks (20%). We found only one patient (2.5%) of the yellow race.

### Presence of the *Agger nasi cell*

The ANC was present in 98.7% of the sides, not being seen in only one side of a patient (right side).

### *Agger nasi cell* measures

#### Anteroposterior diameter of the Agger nasi cell (AGAP)

There was no significant difference in the measures vis-à-vis the side among men and in the total sample, only among women (Table 1).

**Table 1:** Measure in millimeters of the largest anteroposterior diameter of the *Agger nasi cell* (AGAP).

AGAP	Females		Males		All	
	Right	Left	Right	Left	Right	Left
Mean	7.28	5.67	6.48	6.86	6.85	6.31
Standard deviation	2.97	2.47	2.68	2.85	2.81	2.72
Minimum value	3	2	4	3	3	2
Maximum value	12	10	13	14	13	14
*p*	< 0.001		0.428		0.116	

#### Side-to-side diameter of the Agger nasi cell (AGLL)

Table 2 shows the mean values for females, males and total sample. There was a significant difference in the measures vis-à-vis the right and left sides among women and considering the total sample of men and women.

**Table 2:** Measure in millimeters of the largest anteroposterior diameter of the *Agger nasi cell* (AGLL).

AGLL	Females		Males		All	
	Right	Left	Right	Left	Right	Left
Mean	5.72	4.78	6.14	5.71	5.95	5.28
Standard deviation	2.40	2.05	1.77	2.03	2.06	2.06
Minimum value	2	2	3	2	2	2
Maximum value	11	9	9	10	11	10
*p*	0.025		0.369		0.036	

#### Agger nasi cell *Craniocaudal Diameter (AGCC)*

Evaluating the largest cranial-caudal diameter of the *Agger nasi cell* (AGCC), no statistically significant differences were found between the genders and between the right and left sides (Table 3).

**Table 3:** Measure in millimeters of the largest craniocaudal diameter of the *Agger nasi cell* (AGCC).

AGCC	Females		Males		All	
	Right	Left	Right	Left	Right	Left
Mean	6.67	7.39	7.29	6.90	7.00	7.13
Standard deviation	3.20	3.84	1.79	2.32	2.52	3.08
Minimum value	2	2	4	3	2	2
Maximum value	13	14	12	11	13	14
*p*	0.195		0.390		0.714	

### Frontal sinus ostium measures

#### Anteroposterior diameter of the frontal sinus ostium (OFAP)

Table 4 depicts the Anteroposterior frontal ostium (OFAP) values, with no statistically significant difference between the genders and in the total sample. When analyzing the 80 sides, without differentiating for gender, the mean value was 8.13 mm.

**Table 4:** Measure in millimeters of the largest anteroposterior diameter of the frontal sinus ostium (OFAP).

OFAP	Females		Males		All	
	Right	Left	Right	Left	Right	Left
Mean	8.39	7.44	8.00	8.59	8.18	8.08
Standard Deviation	3.33	3.03	2.31	2.34	2.78	2.70
Minimum value	3	3	5	3	3	3
Maximum value	16	13	13	12	16	13
*p*	0.229		0.188		0.816	

#### Side-to-side diameter of the frontal sinus ostium (OFLL)

The mean values of the frontal sinus ostium side-to-side diameter (OFLL) in the total sample was 6.50 mm (SD = 2.17 mm) to the right and of 6.45 mm (SD = 2.05 mm) to the left. Statistically significant difference was not observed between the sides and the genders (Table 5). Considering the 80 sides, the mean value was 6.48 mm.

**Table 5:** Measure in millimeters of the largest side-to-side diameter of the frontal sinus ostium (OFLL).

OFLL	Females		Males		All	
	Right	Left	Right	Left	Right	Left
Mean	6.17	5.94	6.77	6.86	6.50	6.45
Standard Deviation	2.53	1.70	1.85	2.25	2.17	2.05
Minimum value	3	3	4	4	3	3
Maximum value	12	8	12	13	12	13
*p*	0.675		0.835		0.880	

### Gender comparison

As far as gender is concerned, there was no significant difference between the measures evaluated (Table 6).

**Table 6:** Gender comparison (measures in mm).

			Mean	Median	SD	*P*
OFAP	Right	Females	8.39	9	3.33	0.666
		Males	8.00	8	2.31	

	Left	Females	7.44	7	3.03	0.185
Males		8.59	9	2.34		

OFLL	Right	Females	6.17	6	2.53	0.387
		Males	6.77	7	1.85	

	Left	Females	5.94	6	1.70	0.161
Males		6.86	7	2.25		

AGAP	Right	Females	7.28	8	2.97	0.381
		Males	6.48	6	2.68	

	Left	Females	5.67	5	2.47	0.180
Males		6.82	6	2.79		

AGCC	Right	Females	6.67	6	3.20	0.452
		Males	7.29	7	1.79	

	Left	Females	7.39	7	3.84	0.510
Males		6.73	6	2.41		

AGLL	Right	Females	5.72	6	2.40	0.533
		Males	6.14	6	1.77	

	Left	Females	4.78	5	2.05	0.101
Males		5.91	6	2.18		

### Comparison between the races

In order to assess the race influence on the above measures, we made a paired analysis of each one of the measures, comparing the races (white versus black, white versus brown, brown versus black). All measures (AGAPE, AGLLD, AGLLE, AGCCD, AGCCE, OFAPD, OFAPE, OFLLD and OFLLE) showed no statistically significant differences with the exception of AGAPD between the black and brown races, in which we observe a significant difference (*p* = 0.037).

Finally, we compared the three races together (white versus black versus brown), regardless of gender and side, and noticed that there was no significant difference for any of the measures assessed.

### Relation between the *Agger nasi cell* and frontal sinus ostium measures

Analyzing the relationships between the various measures of the ANC and FO through the Pearson correlation, we concluded that they all have a statistically significant positive correlation, but with a bad or very bad correlation rate (Table 7).

**Table 7:** Correlation (R) between the *Agger nasi cell* measures and those from the frontal sinus ostium.

General		OFAP	OFLL	AGAP	AGCC
OFLL	R	44.3%	-	-	-
	*p*	< 0.001	-	-	-

AGAP	R	31.2%	42.0%	-	-
	*p*	0.005	< 0.001	-	-

AGCC	R	38.7%	22.2%	44.8%	-
	*P*	< 0.001	0.050	< 0.001	-

AGLL	R	29.7%	27.5%	36.3%	36.9%
	*p*	0.008	0.014	0.001	0.001

## DISCUSSION

The FR is one of the most complex areas of the paranasal sinuses anatomy, causing difficulties even for the rhinologists who are frequently working in this region. This if justified by the large number of structures that can participate and influence the formation of this communication with the FS, the FO and the FS itself.

Any study that brings some new contribution to the knowledge of this region helps us better understand its anatomy and provides more data for decision-making aimed at the management of patients with sinonasal diseases.

In our study, the first question to be argued is the use of PSCT scan to assess the FR, the FO and the ANC. Most studies in the literature use CT scans to assess these structures, but there is no uniform methodology for such analysis. The type of cross-sectional view (axial, sagittal or coronal) and the slice thickness vary among the studies, making data comparison much too variable. Some authors used only the axial and coronal views for their conclusions^18^, ^19^, ^20^, ^21^, ^22^, ^23^. Although Landsberg et al.^10^ reported that the ANC identification is very difficult even with the use of the sagittal reconstruction, we chose this technique to complement the analysis of the axial and coronal views, since it helped in the identification of the FR, the FO and the ANC, as numerous authors have reported in the literature^7^, ^9^, ^17^, ^24^, ^25^, besides facilitating the measuring of the structures of interest in our study.

One important data of our analysis was the presence or absence of the ANC. Several studies have evaluated the presence of ANC using PSCT scan. We found this cell in 98.7% of the sides studied - such structure was seen only on the left side in one patient. Disregarding the sides, the ANC was found in 100% of our patients. It is worth stressing that the literature data is presented differently, sometimes considering the individual, sometimes the sides to calculate the ANC prevalence. We chose to use the individual-related prevalence, as most of the studies did. In general, the ANC presence varied between 7.77% and 100%. Our study corroborates data from various authors who found the ANC in 95% or more of the patients studied^26^, ^27^. Others found the ANC in about 90% of the patients - very close to the values reported in our study^22^, ^28^. On the other hand, our data is different from that of some authors who lower percentages in their respective studies^20^, ^29^, ^30^.

Analyzing the reason behind such differences, we concluded that the technique used can be an important explanation for the discrepancy found. Some authors used only the CT scan with thicker axial and coronal slices (thickness equal to or larger than 3 mm), getting lower ANC percentages. Since we choose the PSCT with sagittal reconstruction and thin slices (1 mm), this technique proved to be better for ANC identification when compared to the other studies, and we believe it should be the choice of approach to analyze this region. Finally, we found other authors who, even using the sagittal CT reconstruction with thin slices, similar technique to the one used in this study, did not achieve our results^16^, ^31^, ^32^.

Perhaps the size of our sample has been the factor that led to this difference, since the sample of the studies from Landsberg et al.^10^ and Mazza et al.^31^ were larger. Hilger et al.^16^ assessed only ten cases, and this has certainly compromised their final results. The only data that called our attention in the literature review, was the one from Kayalioglu et al.^29^, who found the ANC in only 7.77% of the studied cases. Revising their paper, we noticed that this low prevalence occurred due to the ANC definition they chose, which differs from the regularly used nomenclature^6^ and it is closer to the definition of an ethmoidofrontal cell and not the ANC itself.

The ANC measures, on the other hand, are not frequently reported in the literature: only one study evaluated the anteroposterior measure of the ANC^9^. In our results we found a slightly lower mean value (6.85 mm on the right and 6.31 mm on the left) in relation to the results from this author (9.1 mm on the right and 8.7 mm on the left). We believe that this difference is due to the type of population assessed (Brazilian versus Americans), not being able to discard the influence of the race and physical biotype, as per already demonstrated in another comparative study between different peoples, which assessed, for instance, the ANC presence^20^. We did not find in the literature data on ANC measurements in the side-to-side and craniocaudal directions and our data only points to measures associated to the small population sample and one from a given region, but it can be an incentive and a basis for future studies.

FO size is the object of investigation of some authors and the measures found vary in the literature. All the evaluated studies describe the FO dimensions considering the mean values of the measures of the right and left sides together and not individualizing the nasal cavities. In our study, we differentiated the measures for each side and we also calculated the mean value of the measures for the 80 studied sides in order to better compare our findings and those from the other researchers. In our sample, the OFAP measured 8.13 mm, considering the 80 sides, slightly larger than those found by Landsberg et al.^10^ (mean = 7.22 mm) and DelGaudio et al.^33^, who found the mean value of 7.4 mm. On the other hand, our result was closer to the one found by Farhat et al.^34^ (mean = 7.9 mm). The OFAP measures, both for the right and for the left sides, found in our series were lower (mean = 8.08 mm to left, and 8.18 mm to the right) in relation to those reported by Jacobs et al.^9^ (mean = 10.5 mm in the left and 10.3 mm in the right), with the caveat that the sample used by that author was half of ours (20 patients), and this may have influenced their results. For the OFLL, we found a mean value of 6.48 mm, which was different from that of Landsberg al.^10^ who found a mean value of 8.92 mm for the same measure, but they assessed more patients (144 patients). Although the technique used in the PSCT was the same in our study and in all the other studies cited, once again the sample size and the population diversity could have influenced the values found.

As far as the races are concerned, our sample's characteristic is very similar to the one of the Brazilian population in general (IBGE - Census 2010)^35^. The only studies in which race was considered, involve the ANC prevalence. In our sample, the ANC was present in 100% of the sides for whites, blacks and yellows; and in 92.3% of the sides in browns. Most of the studies involve patients of the yellow race, and corroborate our findings (92.1%^21^; 94%^36^; 94.1%^37^; 95.3%^23^). Only Badia et al.^20^ found a lower prevalence (47%), but their sample was also larger (100 patients). This same author found a 44% prevalence of ANC among Caucasians (100 patients).

In the literature we found no race distinctions in the numerous studies published concerning ANC and FO measures. In our study we did not find statistically significant differences between the measures (AGAP, AGCC, AGLL, OFAP and OFLL) among whites, browns and blacks. We did no statistical assessment of patients from the yellow race, since we had only one patient in that category. The only measure with relevant statistical difference was the AGAPD among blacks and browns. When we compared the measures of the ANC and FO among the races, without considering gender and size, we found no statistically significant differences among them.

Numerous authors report that the ANC block the FR, and such anatomical alteration can be one of the possible causes of frontal sinus disease^6^, ^13^, ^19^, ^26^, ^38^, ^39^. Daniels et al.^40^ stress that the ANC is an important structure that influences the dimensions of the FO and the size of the internal nasal spine, data corroborated by Wormald^4^, who stresses that the ANC pneumatization influences the size of the internal nasal spine and, consequently, the FO size. They all report only qualitative findings derived from the analysis of the anatomical structures in the PSCT scan and their spatial positioning. However, we did not find quantitative studies measuring how much the ANC influences the FO dimensions. In our study, we used the Pearson's correlation in an attempt to quantify how much the ANC measures variation would influence the FO dimensions. We found that all the variables assessed (AGAP, AGLL, AGCC, OFAP e OFLL) had positive correlation, that is, when one increases or decreases, the others also increases or decreases, in a direct ratio, but this is a poor correlation. These findings goes hand-in-hand with Wormald's qualitative findings^13^, who concluded that great pneumatization of the ANC cause smaller internal nasal spines and, consequently, larger frontal ostia. Thus, in our sample we cannot state that the ANC strongly influenced the anteroposterior and side-to-side dimensions of the FO.

## CONCLUSIONS

The *Agger nasi cell* (ANC) prevalence was 98.7% in the nasal cavities and in 100% of the patients studied. There was no statistically significant difference among the mean values of the ANC and FO measures vis-à-vis the total sample. There was no statistically significant difference between the genders and among the races for all the measures. The ANC measures had a poor or very poor correlation with the FO measures.
